# Anti-cyclic citrullinated peptide antibody titer predicts time to rheumatoid arthritis onset in patients with undifferentiated arthritis: results from a 2-year prospective study

**DOI:** 10.1186/ar4148

**Published:** 2013-01-22

**Authors:** Nicola Bizzaro, Elena Bartoloni, Gabriella Morozzi, Stefania Manganelli, Valeria Riccieri, Paola Sabatini, Matteo Filippini, Marilina Tampoia, Antonella Afeltra, Giandomenico Sebastiani, Claudia Alpini, Vittorio Bini, Onelia Bistoni, Alessia Alunno, Roberto Gerli

**Affiliations:** 1 Laboratorio di Patologia Clinica, Ospedale San Antonio, 33028 Tolmezzo, Italy; 2 Struttura di Reumatologia, Dipartimento di Medicina Clinica e Sperimentale, Università di Perugia, 06122 Perugia, Italy; 3 Dipartimento di Scienze Mediche, Chirurgiche e Neuroscienze, Università degli Studi di Siena, 53100 Siena, Italy; 4 Dip. di Medicina Interna e Specialità Mediche, Università Sapienza di Roma, 00185 Roma, Italy; 5 UOC di Patologia Clinica, D.E.A. II Umberto I, 84014 Nocera Inferiore, Italy; 6 Divisione di Reumatologia, Spedali Civili di Brescia, 25125 Brescia, Italy; 7 Laboratorio di Patologia Clinica, Azienda Ospedaliera Universitaria, Policlinico di Bari, 70124 Bari, Italy; 8 U.O.C. di Medicina Clinica e Reumatologia, Università Campus Bio-Medico, 00128 Roma, Italy; 9 U.O.C. di Reumatologia, Azienda Ospedaliera San Camillo - Forlanini, 00151 Roma, Italy; 10 S.C. Analisi Chimico Cliniche, Fondazione IRCCS Policlinico S.Matteo, 27100 Pavia, Italy; 11 Dipartimento di Medicina Interna, Università di Perugia, 06122 Perugia, Italy

## Abstract

**Introduction:**

The diagnostic, predictive and prognostic role of anti-cyclic citrullinated peptide (CCP) antibodies in rheumatoid arthritis (RA) patients is widely accepted. Moreover, detection of these antibodies in subjects presenting with undifferentiated arthritis (UA) is associated with a significant risk to develop the disease. On the other hand, clinical and prognostic significance of evaluating anti-CCP levels in subjects with inflammatory arthritis at disease onset has not been fully clarified. The goal of this prospective study is to analyze the value and prognostic significance of anti-CCP titer quantification in UA subjects.

**Methods:**

Serial anti-CCP assays were measured in 192 consecutive patients presenting with UA lasting less than 12 weeks. Clinical and serological data and arthritis outcome were evaluated every 6 months until two years of follow-up.

**Results:**

Anti-CCP positivity, at both low and high titer, and arthritis of hand joints significantly predicted RA at two years, risk increasing in subjects with high anti-CCP titers at baseline. Moreover, time to RA diagnosis was shorter in patients with high anti-CCP2 titers at enrollment with respect to those with low antibody concentration.

**Conclusions:**

Presence of anti-CCP antibodies, at both low and high concentration, is significantly associated with RA development in subjects with recent onset UA. However, time interval from the onset of the first symptoms to the fulfilment of the classification criteria appears to be directly related to the initial anti-CCP level.

## Introduction

In recent years, the broad availability of specific serological markers deeply changed the diagnostic approach to rheumatoid arthritis (RA), a chronic inflammatory disease associated with a progressive and often disabling course if not promptly recognized and effectively treated. In this setting, anti-citrullinated peptide antibodies (ACPA), commonly detected by means of the second generation anti-cyclic citrullinated peptide test (anti-CCP2), represent a peculiar feature of RA patients [1]. To meet the need of improved diagnostic and prognostic tests, the progressive evolution of the analytical methods for ACPA detection, as measured by quantitative immunometric assays, have led to a very high level of diagnostic accuracy with a specificity of 95% to 97% and a sensitivity of 67% to 80% [2,3]. The sensitivity values are likely the highest obtainable in relation to the close link existing between the production of ACPA and genetic constitution [4]. At the moment, the anti-CCP2 antibody test yields higher specificity and comparable or even higher sensitivity with respect to rheumatoid factor (RF) or other ACPA, including the recently discovered anti-mutated citrullinated vimentin antibodies [5].

In established disease, it is has been widely demonstrated that the presence, in particular at high levels, of anti-CCP is associated with more severe clinical outcomes, higher disease activity and worse radiographic progression [6-9]. Moreover, retrospective studies have assessed their predictive value demonstrating that anti-CCP can be detected in the serum of subjects later developing RA up to fourteen years before the first clinical symptoms, with titer significantly increasing closer to disease onset [10,11]. Similar findings have been obtained in studies involving patients with early disease, thus confirming the clinical utility of anti-CCP as a diagnostic and prognostic tool in subjects presenting with RA lasting less than one or two years [1,5]. As a consequence, the new 2010 RA Classification Criteria, which have been updated in order to diagnose RA in an earlier phase, included detection of ACPA as a key item for diagnosing the disease [12].

Finally, anti-CCP antibodies may have an important role in the diagnostic algorithm of subjects presenting with undifferentiated arthritis (UA). Indeed, UA accounts for 30% to 50% of patients presenting to the rheumatologist and has a variable natural course. In particular, progression to RA has been reported in only one-third of patients after 1 year and in 40% after 3 years [13]. In order to minimize the risk of diagnostic pitfalls and subsequent under- or over-treatment, clinical, serologic and instrumental markers have been employed to estimate the likelihood of progression to RA in these subjects. Among these, serum anti-CCP positivity at baseline has been demonstrated to possess very high predictive and prognostic accuracy in comparison to other markers [14]. Interestingly, a recent study showed that early introduction of methotrexate therapy in UA patients with circulating anti-CCP delays evolution to RA, and prevents joint damage [15].

Although some questions remain unanswered regarding the significance of anti-CCP detection in patients with UA, quantification of anti-CCP serum level is now considered a key investigational issue and the important role played by antibody level on disease outcome has been underlined by the different scores attributed to antibody serum levels in the new classification criteria for RA [12]. Indeed, in early RA lasting less than 1 year, it has been demonstrated that anti-CCP positivity at any time is associated with higher risk of radiographic damage at baseline [16]. Interestingly, increase in antibody titer during the first 3 years of follow up was shown to significantly correlate with radiographic progression after 5 years [16]. However, in a similar patient population, anti-CCP serum levels did not seem to correlate with disease activity and severity, thereby suggesting that anti-CCP-positive patients with early RA have higher disease activity and severity independent of antibody titer [17].

On the other hand, studies analyzing the value and prognostic significance of anti-CCP titer quantification in UA patients at disease onset are very few, and results quite contradictory, mainly because of different study design and population enrollment criteria. In UA patients with evidence of circulating anti-CCP at disease onset, it has been demonstrated that antibody status appears to remain substantially stable during a disease course of up to 5 years of follow up, with a rate of seroconversion ranging from 1% to 9% [18-21]. Thus, the utility of anti-CCP retesting during the disease course in patients presenting with inflammatory arthritis is actually questionable and not recommended. On the other hand, the clinical value of anti-CCP levels at disease onset and influence of titer changes over time on disease outcome have not been fully clarified.

Therefore, the aim of this multicenter prospective study was to analyze the value and prognostic significance of anti-CCP titer quantification and monitoring in a cohort of patients presenting with UA.

## Material and methods

### Study subjects

Consecutive patients with UA were recruited at nine rheumatology units belonging to the Forum Interdisciplinare per la Ricerca nelle Malattie Autoimmuni (FIRMA group), an Italian association of hospital and university experts in the field of autoimmune rheumatic diseases. The cohort included patients aged more than 18 years presenting with either mono-, oligo- or polyarticular arthritis lasting less than 12 weeks, and not meeting the 1987 American College of Rheumatology (ACR) classification criteria for RA, nor fulfilling any of the existing classification criteria for other inflammatory rheumatic disease. Evidence of radiographic joint damage or rheumatoid nodules represented exclusion criteria. Only non-steroidal anti-inflammatory drugs (NSAIDs) or Cox-2 selective inhibitors (Coxibs) and/or low-dose of corticosteroids (CS) (prednisone < 15 mg a day or equivalent) were allowed in the period from symptom onset to the time of enrollment. Patient recruitment began in March 2007 and was stopped in May 2009 after the inclusion of 206 patients. Subjects were assessed by a trained rheumatologist at baseline and every 6 months thereafter. Follow-up was continued until the last enrolled patient completed two years of follow-up. Demographic data were assessed at baseline and clinical and serological data were recorded at each visit. Diagnosis of RA or of any other rheumatic or non-rheumatic disorder was performed by the referring rheumatologists.

Each enrolled patient gave written consent prior to being included in the study. Patient identity was not disclosed and the data were anonymously used in accordance with the latest version of the Helsinki Declaration of human research ethics. Collection of patient samples was carried out according to the University-Hospital of Udine Ethic Committee regulations.

### Methods

Serum samples were collected at baseline and every 6 months. Overall, therefore, four serum samples were obtained from each patient. ACPAs were measured by the routine commercial method used in each participating center, according to manufacturer's instructions. They were determined by CCP2-based assays, manufactured by Phadia (Uppsala, Sweden) (three centers), Axis-Shield (Dundee, UK) (three centers), Eurodiagnostica (Nijmegen, The Netherlands) (two centers) and Inova (San Diego, CA, USA) (one center). To harmonize and compare results obtained by the different assays, anti-CCP2 levels were expressed as a ratio, dividing the observed absolute antibody amount by the cutoff value of each commercial kit. Serum levels less than, or more than three times the cutoff were considered low and high, respectively.

IgM RF, erythrocyte sedimentation rate (ESR) and C-reactive protein (CRP) levels were locally determined by each participating center. Since the nine centers employed different analytical methods with different cutoffs, RF, ESR and CRP values were also converted to ratios, as described for anti-CCP2 values.

### Statistical analysis

Student's *t*-test and the Mann-Whitney test were used to compare normally and non-normally distributed continuous variables respectively (deviation from the Gaussian distribution were checked using the Shapiro-Wilk test). The chi squared (X^2^) test with Yate's continuity correction was used to analyze categorical variables. To estimate the survival function from the time of arthritis onset to the time of RA diagnosis, the Kaplan-Meier method was used. The risk of developing RA according to CRP, RF, anti-CCP2, and arthritis of the hand joints, was estimated by the Cox proportional hazard model, first adding one variable at time (un-adjusted hazard ratio, HR), and then all variables together (adjusted HR).

Biochemical variables were evaluated as categorical variables and divided into three levels as suggested by the ACR/European League Against Rheumatism (EULAR) criteria. Two-tailed *P*-values lower than 0.05 were considered statistically significant. Calculations were performed with Stata 8.2 software (Stata Corporation, College Station, Texas, USA).

## Results

Among the 206 enrolled patients, 192 (93.2%) completed the study and 14 were lost during follow up, mainly because they moved to another location. Table 1 shows the baseline characteristics of the patient cohort. It includes 147 women and 45 men with a mean age of 52 ± SD, 16 years. Most patients had polyarthritis (60.9%) and only 9.4% of them presented with monoarthritis at onset. In about two-thirds of the cases, joint synovitis was localized at the hands.

**Table 1:** Baseline demographic, clinical and serological characteristics of the 192 patients with undifferentiated arthritis

Characteristics	Baseline values	Percent patients
Age, years, mean ± SD	52 ± 16	na
Female, n	147	76.6
Monoarthritis, n	18	9.4
Oligoarthritis, n	57	29.7
Polyarthritis, n	117	60.9
Arthritis of hand joints, n	126	65.6
CRP		
Positive, n	98	51.0
Ratio, median (IR)	1.1 (0.6-4.1)	na
ESR		
Increased, n	103	53.6
Ratio, median (IR)	22.5 (14-40)	na
Rheumatoid factor		
Positive	79	(41.4)
Ratio, median (IR)	0.7 (0.5-3.9)	na
Low titer on total positive	26	(32.9)
High titer on total positive	53	(67.1)
ACPA		
Positive, n	80	(41.7)
Ratio, median (IR)	0.5 (0.3-7.8)	na
Low levels on total positive, n	13	(16.2)
High levels on total positive, n	67	(83.8)
Baseline therapy		
None, n	88	(45.8)
NSAIDs/Coxibs, n	66	(34.4)
Corticosteroids, n	10	(5.2)

n, number of patients; CRP, C-reactive protein; IR, interquartile range; ESR, erythrocyte sedimentation rate; ACPA, anti-citrullinated peptide antibodies; NSAID, non-steroidal anti-inflammatory drug; Coxib, Cox-2 selective inhibitor; na, not applicable.

A consistent number of patients (*n *= 79, 41.4%) were positive for RF (mean ratio: 3.8 ± SD 8.2) and/or anti-CCP2 (*n *= 80, 41.7%; mean ratio 7.8 ± SD 17) at baseline. Among the RF-positive subjects, 67.1% displayed high RF titers, while in the group of anti-CCP2-positive subjects, an even higher percentage (83.8%) displayed high anti-CCP2 levels in the serum. Many patients (45.8%) were out of therapy, 66 patients (34.4%) were taking NSAIDs or Coxibs, while a small number of subjects were taking CS in combination with, or without NSAIDs/Coxibs.

As depicted in Table 2, 72/192 (37.5%) patients had progressed to RA at 2 years from recruitment: 26 at 6 months, 20 at 12 months and 26 at 24 months. Among those who did not develop RA, a rheumatic disease other than RA (largely psoriatic arthritis and undifferentiated connective tissue disease) was diagnosed in 30 patients (15.6%). In addition, arthritis was transitory and eventually remitted in 32 patients (16.7%), while no definite diagnosis was made at 24 months in 58 of them (30.2%) and a diagnosis of UA was thus maintained.

**Table 2:** Diagnosis at the end of follow up (2 years)

Diagnosis	Number of patients (%)
Rheumatoid arthritis	72 (37)
Undifferentiated arthritis	58 (30)
Remission	32 (17)
Psoriatic arthritis	11 (6)
Undifferentiated connective tissue disease	8 (4)
Fibromyalgia	4 (2)
Spondiloarthritis	2 (1)
Viral arthritis (EBV)	1 (0.5)
Systemic lupus erythematosus	1 (0.5)
Osteoarthritis	1 (0.5)
Sarcoidosis	1 (0.5)
Primary Sjögren's syndrome	1 (0.5)
Total	192 (100)

EBV, Epstein-Barr virus.

Table 3 shows the baseline characteristics of the patient cohort who developed RA at the end of the study, compared to the patients who did not. Age, gender and number of involved joints at presentation did not help in distinguishing patients developing RA during follow up, while hand articular involvement was more frequent at baseline in subjects developing RA. Moreover, higher initial levels of CRP but not of ESR, were associated with a higher possibility of developing RA. In addition, the majority of patients with a diagnosis of RA at the end of the study were RF-positive and had much higher titers of RF at presentation than patients who did not develop the disease. However, the percentage of patients with either low or high RF titer at symptom onset in RA and non-RA patients was similar.

**Table 3:** Baseline demographic, clinical and serological characteristics of the 192 patients subdivided according to diagnosis after 24 months

Parameter at onset	Patients developing RA		Patients not developing RA		*P*
		
	Number	%	Number	%	
Patients	72	37.5	120	62.5	
Age, years, mean ± SD	52 ± 16	na	52 ± 15	na	0.895
Female	57	79.2	90	75.0	0.628
Monoarthritis	7	9.7	11	9.2	0.999
Oligoarthritis	16	22.2	41	34.2	0.111
Polyarthritis	49	68.1	68	56.6	0.158
Hand joints arthritis	55	76.4	71	59.2	**0.023**
CRP+	41	56.9	58	48.3	0.314
CRP ratio, median (IR)	1.9 (0.6-1.9)		0.9 (0.6-2.6)		**0.031**
ESR increase	38	52.8	65	54.2	0.970
ESR median (IR)	24 (12.3-53.3)		22.5 (16.0-38)		0.773
RF+	45	62.5	34	28.3	**< 0.0001**
RF ratio, median (IR)	2.3 (0.6-7.8)		0.6 (0.5-1.2)		**< 0.0001**
RF low+/total RF+	11	24.4	15	44.1	0.109
RF high+/total RF+	34	75.5	19	55.9	
ACPA+	53	73.6	27	22.5	**< 0.0001**
ACPA ratio, median (IR)	5 (0.6-14.3)		0.5 (0.3-0.8)		**< 0.0001**
ACPA low+/total+	7	13.2	6	22.2	0.476
ACPA high+/total+	46	86.8	21	77.8	
RF+/ACPA-	2	2.8	12	10	0.115
RF-/ACPA+	10	13.9	5	4.2	**0.031**
RF+/ACPA+	43	59.7	21	18.3	**< 0.0001**
RF-/ACPA-	17	23.6	81	67.5	**< 0.0001**

RA, rheumatoid arthritis; CRP, C-reactive protein; IR, interquartile range; ESR, erythrocyte sedimentation rate; RF, rheumatoid factor; ACPA, anti-citrullinated peptide antibodies; NA, not applicable.

Among subjects who developed RA, the majority had anti-CCP2 at presentation (73.6%), while only 22.5% of non-RA patients displayed these antibodies (*P *< 0.0001). Interestingly, although the anti-CCP2 mean ratio at baseline in the RA cohort was up to 3.5-fold higher than that observed in the non-RA group (*P *< 0.0001), the percentage of patients with either low or high anti-CCP2 titers at enrollment did not differ in the RA and non-RA groups. Furthermore, the presence at recruitment of a single positivity for RF, without evidence for anti-CCP2, was not able to discriminate between subjects who would or would not develop RA, while the percentage of anti-CCP2-positive patients was higher in the RA cohort independent of the evidence of RF-positivity. It is of note that the percentage of anti-CCP2-positive patients and anti-CCP2 titers remained substantially stable during follow up (data not shown).

Finally, the initial treatment with NSAIDs/Coxibs and/or CS did not appear to influence the subsequent development of arthritis (data not shown).

On univariate regression analysis, inflammatory involvement of the hand joints, high CRP levels, and RF and anti-CCP2 at both low and high titers were predictive of RA development (Table 4). However, on multivariate analysis adjusted for the covariates CRP, RF, anti-CCP2 and arthritis of the hand joints, only arthritis of the hand joints and anti-CCP2, at both low and high titers predicted RA, with a risk of 3.2 for low, and 4.3 for high anti-CCP2 titers at baseline.

**Table 4:** Risk of developing rheumatoid arthritis: Cox regression univariate and multivariate hazards ratio (HR) analysis

	HR (95% CI) Unadjusted	*P*	HR (95% CI) Adjusted	*P*
**CRP**				
**Negative**	1		1	
**Low +**	0.720 (0.362, 1.434)	0.350	0.875 (0.423, 1.811)	0.719
**High+**	1.737 (1.049, 2.877)	0.032	1.827 (0.950, 3.514)	0.071
**RF**				
**Negative**	1		1	
**Low+**	2.020 (1.001, 4.073)	0.050	0.791 (0.248, 2.522)	0.692
**High+**	3.097 (1.867, 5.137)	< 0.001	0.729 (0.219, 2.431)	0.607
**ACPA**				
**Negative**	1		1	
**Low +**	3.360 (1.412, 7.998)	0.006	3.187 (1.257, 8.077)	0.015
**High +**	4.613 (2.698, 7.887)	< 0.001	4.324 (2.023, 9.245)	< 0.001
**Hand joints arthritis**	1.871 (1.083, 3.232)	0.025	2.140 (1.128, 4.059)	0.020

CRP, C-reactive protein; RF, rheumatoid factor; ACPA, anti-citrullinated peptide antibodies.

**Figure 1:**
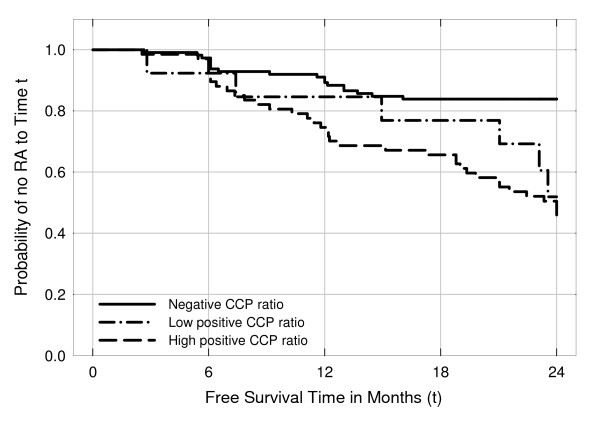
**Time to reach rheumatoid arthritis (RA) diagnosis according to anti-cyclic citrullinated peptide 2 (CCP2) levels in 192 patients with undifferentiated arthritis**. Data were analyzed by Kaplan-Meier analysis and the log-rank test.

As shown in Figure 1, time from presentation to diagnosis of RA was related to anti-CCP2 levels at baseline, since this period was shorter in patients having high anti-CCP2 titers at enrollment with respect to those displaying basal low anti-CCP2 levels. However, the RA-free survival curve in the two groups overlapped at the end of 2-year follow up.

During the study period, patients were treated with anti-inflammatory drugs, or disease modifying anti-rheumatic drugs (DMARDs), but there was no relationship between the change in anti-CCP2 levels and the therapy introduced (data not shown). Moreover, no significant relationship was detected between anti-CCP2 titer and the other parameters, in particular ESR and CRP (data not shown).

## Discussion

In recent years, the generation of serological tests for accurate ACPA detection lead to an impressive improvement in the diagnosis of inflammatory articular disorders. In particular, anti-CCP2 assays are helpful in various clinical settings, such as early diagnosis of RA, diagnosing RF-negative RA and differentiating other RF-positive arthritis, such as hepatitis C virus-related joint involvement [1,22]. Moreover, in subjects presenting with UA, evidence of anti-CCP2 positivity at baseline appears to have relevant diagnostic, predictive and prognostic value [16,23-25]. On the other hand, major uncertainty remains about the clinical and prognostic significance of anti-CCP2 titer in UA subjects, in particular in subjects presenting with borderline antibody values. These doubts are further strengthened by the observation that many non-RA sera with low ACPA levels bind equally to citrullinated and non-citrullinated peptides and that low ACPA levels may not discriminate between true and false positive results [26]. Thus, specificity of low-positive samples remains a diagnostic issue.

In the present prospective study recruiting subjects with recent onset UA, 37% of them developed RA at 2 years, while 30% maintained persistent UA. The available data in the literature show that the percentage of UA subjects developing RA or persistent arthritis is highly variable, ranging from 7% to 72%. The wide range of RA diagnoses may be related to different lengths of follow up, which in particular, was shorter in many studies with respect to that of our investigation [27-30]. Indeed, it is conceivable that some UA patients may experience progression to RA after more than 1 year of follow up. In addition, the design of some studies also did not rule out baseline recruitment of subjects fulfilling the ACR 1987 RA classification criteria. The introduction of DMARDs in this group of patients at enrollment may hamper the interpretation and comparison of the data [21,29,31]. Finally, the pattern of inflammatory joint involvement, and most importantly, the duration of joint symptoms at inclusion ranging from more than four weeks to less than 3 years, produced quite variable differences in results between different studies. In this setting, the very short duration of UA, the exclusion of patients with definite arthritis and the inclusion of all patterns of arthritis, which characterize the enrolled population, may represent a potential strength of this study.

The analysis of clinical and serological characteristics of our UA patients at presentation showed that those developing RA at 2 years more frequently had inflammatory involvement in the hand and higher CRP, RF and anti-CCP2 titers in comparison to non-RA subjects. Although the same items predicted RA on univariate analysis, only patients with hand arthritis and anti-CPP2 positivity presented a significantly increased risk of developing RA on multivariate analysis.

The percentage of anti-CCP2-positive UA patients included in the present study was within the range of anti-CCP2 positivity described in UA cohorts in other studies (14% to 59%). In this setting, it is of note that the proportion of anti-CCP2-positive patients at baseline was higher in subjects with RA diagnosis with respect to non-RA subjects, independent of RF positivity, while the percentage of patients with single positivity for RF (anti-CCP2-negative) was similar in the RA and non-RA groups. Moreover, CRP and ESR levels do not appear to have an effect on risk of progression to RA. Taken together, these observations confirm the key prognostic role of anti-CCP2 for RA diagnosis, rather than RF and inflammatory parameters.

In agreement with previous studies [21,32], we did not observe any effect of age and gender on anti-CCP2 levels in the different patient groups. On the other hand, the genetic background may exert a relevant adjunctive role in the risk of disease development. Interestingly in fact, anti-CCP2-positive UA patients who will develop RA display reactivity against a significant larger number of citrullinated epitopes, namely vimentin, fibrinogen and α-enolase, with respect to anti-CCP2-positive patients not evolving toward RA at one year of follow up, thus postulating a very distinct immunological reactivity profile at disease onset [33].

Recent prospective studies showed that higher baseline anti-CCP titer is significantly correlated to increased likelihood of persistent arthritis or RA development in cohorts of subjects presenting with UA [21,27,29,30] (Table 5). Moreover, the likelihood of persistent arthritis increased with increasing ACPA levels, with a 14-fold risk depicted in subjects with anti-CCP > 250 U/ml [29]. Few of these studies, however, performed a serial analysis of anti-CCP2 titer during follow up, as was performed in our population [21,31]. In the present cohort, the percentage of patients with low or high anti-CCP2 titer was not different in the RA and non-RA groups after 2 years of observation. This reflects the substantial stability of anti-CCP2 levels over time, as has also been demonstrated in other studies [18,19,21].

**Table 5:** Studies evaluating the significance of anti-CCP titer in patients with undifferentiated arthritis (UA)

Author [ref]	Patients, n	UA	UA duration	FU	Anti-CCP	CCP+ baseline	UA outcome	Anti-CCP titer significance
Kudo-Tanaka [27]	146	≥ 2 Sj	≤ 2 yr	1 yr	≥ 5 U/ml no serial assay	17%	12% RA 37% non-RA 41% UA	164 ± 136 RA vs 55 ± 72 non-RA/UA *P *= 0.017
Guzian [18]	253 (83% RA baseline)	≥ 3 Sj	≤ 1 yr	30 mo.	≥ 20 U/ml serial assay (0.30 mo.)	38%	17% RA	No correlation low-high CCP/DAS28, HAQ, erosions
Ursum [31]	545	≥ 2 Sj	≤ 3 yr	2 yr	≥ 5 U/ml serial assay (0.1 yr)	56%	63% RA baseline or at 1 yr	No correlation CCP change/DAS28, HAQ, SHS
Emad [28]	69	≥ 1 Sj	≤ 1 yr	1 yr	≥ 2.9 U/ml no serial assay	59%	26% RA 6% PsA 41% UA 26% remission	Correlation CCP titer/Sj, Tj, ESR, erosions
Bos [30]	147	arthralgia	12 (7-36) mo. median	28 (19-39) mo. median	≥ 5 U/ml no serial assay	34%	7% RA 13% ≥ 4Sj 80% no-arthritis	Median 141 arthritis vs 31 U/ml non-arthritis HR = 1.7
Mjaavatten [29]	376 (19% RA baseline)	≥ 1 Sj	≤ 16 wk	1 yr	≥ 25 U/ml no serial assay	16%	46% persistent 54% self-limiting	25-100 OR 4.4 101-250 OR = 9.4 > 250 OR = 14 for persistence
Burr [21]	640 (49% RA baseline)	≥ 2 Sj	≥ 4 wk	5 yr	≥ 5 U/ml serial assay (0.5 yr)	30%	72% RA 28% non-RA	Median 1.6 U/ml RA vs 0.8 U/ml non-RA
Present study	192	≥ 1 Sj	≤ 12 wk	2 yr	serial assay (0.6,12,18,24 mo.)	42%	37% RA 30% UA 17% self-limiting 16% non-RA RD	Correlation titer/time to RA onset and RA development

RA, rheumatoid arthritis; FU, follow up; Sj, swollen joint; CCP, cyclic citrullinated peptide; Tj, tender joint; DAS28, disease activity score; HAQ, health assessment questionnaire; PsA: psoriatic arthritis; RD, rheumatic disease; SHS, Sharp Heijde Score; ESR, erythrocyte sedimentation rate; HR, hazard ratio; OR, odds ratio.

One of the most interesting findings of the present study, however, is the demonstration that progression to RA in UA patients is more rapid in those with higher anti-CCP2 levels. To our knowledge, this is the first demonstration that high anti-CCP2 levels at baseline correlate with shorter time to RA diagnosis in subjects with recent onset UA. This conclusion appears to be supported by the data of a differently designed study in a cohort of healthy women, showing that detection of high anti-CCP2 levels in samples stored at baseline was strongly associated with the time to RA diagnosis, higher values being predictive of shorter time to disease onset [34].

Anti-CCP concentration does not appear to correlate with clinical disease outcomes or radiographic progression, in particular in studies analyzing serial antibody measurements over time. Neither low nor moderate to high anti-CCP titer, or relative change in antibody levels, correlated with higher risk of radiographic damage, or with outcome measures of disease activity and severity [18,31]. However, in a recent prospective study with a follow up of 5 years, anti-CCP concentration more than four times the upper normal limit was associated with a ten-fold increased risk of erosive disease [21]. The longer follow up characterizing this study may explain these conflicting data.

## Conclusions

The results of the present study further support the value of testing anti-CCP antibodies in subjects presenting with UA. In particular, they confirmed that recent onset UA patients displaying anti-CCP antibodies have a significantly increased risk of developing RA rather than non-RA inflammatory/autoimmune diseases at 2 years. This is confirmed not only in patients with high, but also those with low antibody titer. However, initial anti-CCP levels appear to be of great importance in predicting the interval time to disease onset, since a delay in RA diagnosis could occur in subjects with low antibody levels at symptom onset. This suggests the need of closer follow up of these UA patients. Further studies, however, are needed to evaluate the influence of a peculiar genetic background on antibody level and to explore therapeutic interventions that can be directed toward specific patient subgroups considered to have the highest risks.

## Abbreviations

ACPA: anti-citrullinated peptide antibodies; ACR: American College of Rheumatology; CCP2: cyclic citrullinated peptide 2; Coxibs: Cox-2 selective inhibitors; CRP: C-reactive protein; CS: corticosteroids; DMARDs: disease modifying anti-rheumatic drugs; ESR: erythrocyte sedimentation rate; EULAR: European League Against Rheumatism; FIRMA: Forum Interdisciplinare per la Ricerca nelle Malattie Autoimmuni; HLA: human leukocyte antigen; HR: hazard ratio; NSAIDs: non-steroidal anti-inflammatory drugs; RA: rheumatoid arthritis; RF: rheumatoid factor; SE: shared epitope; UA: undifferentiated arthritis.

## Competing interests

The authors declare that they have no competing interests.

## Authors' contributions

NB conceived the study, designed the study, designed the data collection tools, organized the collaboration, monitored data collection, drafted and revised the manuscript. EB and RG designed the study, drafted and revised the manuscript. GM and SM conceived the study, designed the study, organized the collaboration, contributed to data collection and revised the manuscript. GS and VR contributed to study design, organized the collaboration, contributed to interpretation of findings, preparation and revision of the manuscript. PS, MF, MT, AAf., OB, AAl., and CA contributed to data collection and revised the manuscript. VB performed statistical analysis, drafted and revised the manuscript. All authors have read and approved the final manuscript for publication.
